# Combined bacterial and fungal targeted amplicon sequencing of respiratory samples: Does the DNA extraction method matter?

**DOI:** 10.1371/journal.pone.0232215

**Published:** 2020-04-28

**Authors:** Cécile Angebault, Mathilde Payen, Paul-Louis Woerther, Christophe Rodriguez, Françoise Botterel

**Affiliations:** 1 Unité de Parasitologie-Mycologie, Département de Prévention, Diagnostic et Traitement des Infections, CHU Henri Mondor, Assistance Publique des Hôpitaux de Paris (APHP), Créteil, France; 2 EA DYNAMiC 7380, Faculté de Santé, Univ Paris-Est Créteil, Créteil, France; 3 EA DYNAMiC 7380, Ecole nationale vétérinaire d’Alfort, USC Anses, Maison-Alfort, France; 4 Unité de Bactériologie-Hygiène, Département de Prévention, Diagnostic et Traitement des Infections, CHU Henri Mondor, APHP, Créteil, France; 5 Next-Generation Sequencing Platform”Génomiques”, INSERM U955, APHP, IMRB Créteil, Créteil, France; Louisiana State University, UNITED STATES

## Abstract

**Background:**

High-throughput sequencing techniques are used to analyse the diversity of the respiratory microbiota in health and disease. Although extensive data are available regarding bacterial respiratory microbiota, its fungal component remains poorly studied. This is partly due to the technical issues associated with fungal metagenomics analyses. In this study, we compared two DNA extraction protocols and two fungal amplification targets for combined bacterial and fungal targeted amplicon sequencing analyses of the respiratory microbiota.

**Methods:**

Six sputa, randomly selected from routine samples in Mondor Hospital (*Creteil*, *France*) and treated anonymously, were tested after bacterial and fungal routine culture. Two of which were spiked with *Aspergillus Fumigati* and *Aspergillus Nigri* (10^5^ conidia/mL). After mechanical lysis, DNA was extracted using automated QIAsymphony® extraction (AQE) or manual PowerSoil® MoBio extraction (MPE). DNA yield and purity were compared. DNA extracted from spiked sputa was subjected to (i) real-time PCR for *Aspergillus* DNA detection and (ii) combined metagenomic analyses targeting barcoded primers for fungal ITS1 and ITS2, and bacterial V1-V2 and V3-V4 16S regions. Amplicon libraries were prepared using MiSeq Reagent V3 kit on Illumina platform. Data were analysed using PyroMIC© and SHAMAN software, and compared with culture results.

**Results:**

AQE extraction provided a higher yield of DNA (AQE/MPE DNA ratio = 4.5 [1.3–11]) in a shorter time. The yield of *Aspergillus* DNA detected by qPCR was similar for spiked sputa regardless of extraction protocol. The extraction moderately impacted the diversity or relative abundances of bacterial communities using targeted amplicon sequencing (2/43 taxa impacted). For fungi, the relative abundances of 4/11 major taxa were impacted and AQE results were closer to culture results. The V1-V2 or V3-V4 and ITS1 or ITS2 targets assessed similarly the diversity of bacterial and fungal major taxa, but ITS2 and V3-V4 detected more minor taxa.

**Conclusion:**

Our results showed the importance of DNA extraction for combined bacterial and fungal targeted metagenomics of respiratory samples. The extraction protocol can affect DNA yield and the relative abundances of few bacterial but more fungal taxa. For fungal analysis, ITS2 allowed the detection of a greater number of minor taxa compared with ITS1.

## Introduction

The human respiratory microbiota is a complex ecosystem extending from the nasopharyngeal cavities to the alveoli. It comprises a vast community of microorganisms distributed with relatively variable gradients depending on the site [1]. In this ecosystem, bacteria (e.g. *Streptococcus*, *Veillonella* or *Prevotella*) are the most represented besides fungi, viruses, and archaea. With the development of high throughput sequencing enabling 16S/ITS targeted amplicon high-throughput sequencing and metagenomics, the bacterial component of human respiratory microbiota has become under increasing scrutiny to understand its role in health and disease [1–5]. Lately, it has become evident that the fungal microbiota (mycobiota) should be concomitantly studied to better understand trans-kingdom interactions and the role of fungi in the respiratory pathogenesis. A prerequisite to obtain comparable representations of the microbial communities using high-throughput sequencing methods is the use of appropriate protocols, especially for DNA extraction. The impact of extraction on DNA yield or quality and on community composition has been evaluated in several studies focusing mainly on the digestive bacterial microbiota [6–11]. However, little is known regarding extraction protocols appropriate for fungal analysis or combined bacterial and fungal analyses, especially in case of respiratory microbiota [6,8,12–15]. The technical issues associated with fungal high-throughput sequencing analysis arise from the scarce presence of fungi in the biosphere and from the complex nature of their cell wall [16]. The latter is composed of a thick layer of chitin, beta-glucans, lipids, and peptides with sometimes additional melanin [16], which protects the fungal cell from enzymatic or chemical lysis (an essential step in DNA extraction) [17]. Another issue concerns the choice of the DNA amplification target. Most mycobiota studies have targeted the internal transcribed spacer (ITS) region, as recommended by the Fungal Barcoding Consortium [18], and most authors chose ITS1 [14,15,19–21] rather than ITS2 region [2,3]. Deciding which is better remains uncertain, though ITS1 database is broader than that of ITS2 [14,22]. In the present study, we compared the impact of two recent extraction protocols: the commonly used manual PowerSoil® MoBio extraction (MPE), and the automated QIAsymphony extraction (AQE) using DSP DNA Midi kit, on the fungal and bacterial diversity where both communities were concomitantly assessed via 16S/ITS targeted amplicon sequencing in respiratory samples. We also compared the diversity of (i) fungal community using amplification with ITS1 or ITS2 regions and of (ii) bacterial community using V1-V2 or V3-V4 16S regions. As 16S/ITS targeted amplicon sequencing and culture analyses are rarely performed concomitantly, although it seems essential in a medical laboratory context, we also compared sequencing with culture results.

## Materials and methods

### Sample collection, culture

**Fig 1:**
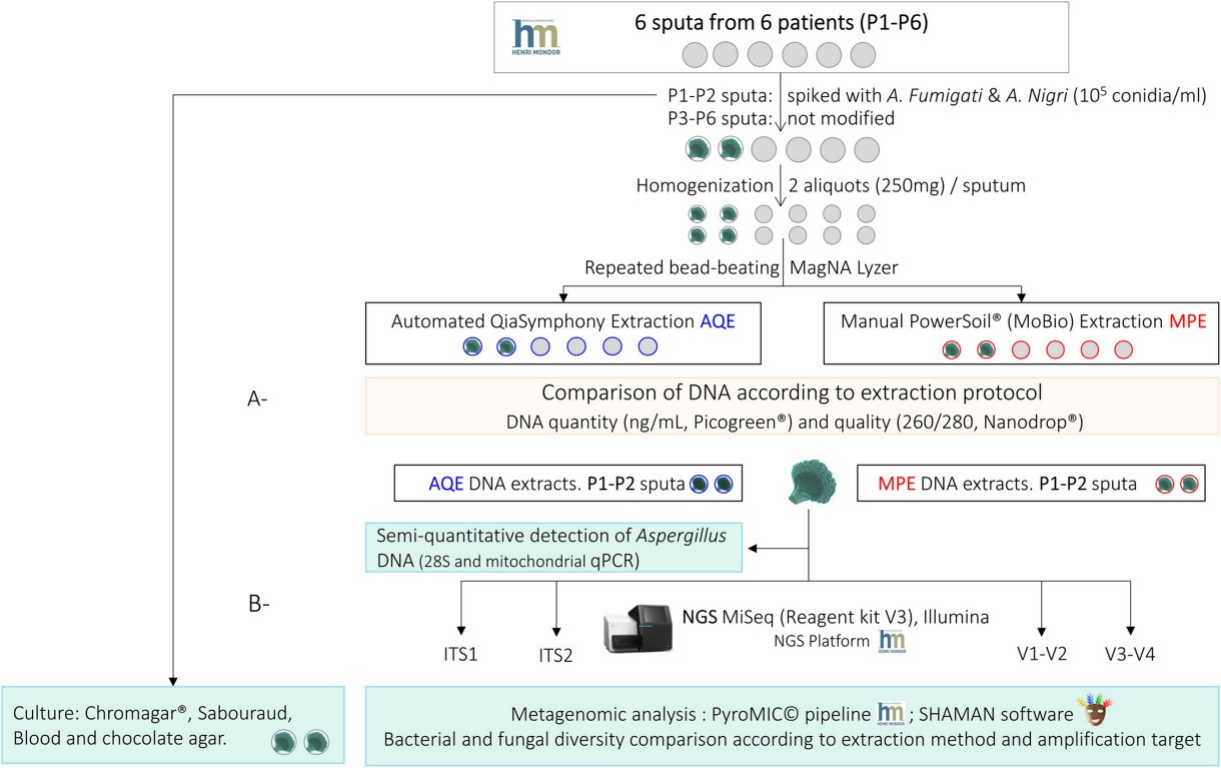
Workflow of the study. A- Comparison of two DNA extraction protocols (automated QIAsymphony extraction [AQE] using DSP DNA midi kit and manual PowerSoil® MoBio extraction [MPE]) in terms of quantity and quality of DNA extracted from 6 sputa (6 patients, P1, P2, P3, P4, P5, P6). B- Comparison of bacterial and fungal diversity detected in two sputa (2 patients, P1, P2) spiked with 10^5^ conidia of *Aspergillus* section *Fumigati* and *Aspergillus* section *Nigri* using (i) culture method and (ii) 16S/ITS targeted amplicon sequencing (DNA extracted using AQE or MPE protocols; amplification targets V1-V2 and V3-V4 16S for bacteria, and ITS2 and ITS2 for fungi; MiSeq® Illumina, 2x300 v3 kit).

Six sputa from patients (P1, P2, P3, P4, P5, and P6) hospitalized in *Henri Mondor* University Hospital (*Créteil*, *France*) were randomly selected from sputa addressed to the microbiological laboratory after assessment of their quality according to Murray and Washington criteria [23]. As part of routine bacterial analysis, they were inoculated onto Chocolate agar + PolyViteX, Columbia agar + 5% horse blood, Columbia ANC agar + 5% sheep blood, Trypicase Soy Agar and Drigalski agar (*bioMérieux*, *Marcy L’Etoile*, *France*) for 48h to 5 days at 35°C. After inclusion in our study, two sputa (P1 and P2) were spiked with 10^5^ conidia of *Aspergillus* section *Fumigati* and 10^5^ conidia of *Aspergillus* section *Nigri*. Ten microliters of the two spiked-sputa (P1 and P2) were inoculated onto two Sabouraud-Chloramphenicol-Gentamicin slants (*Bio-rad*, *Marnes-la-Coquette*, *France*) and onto one BBL^TM^ CHROMagar^TM^ *Candida* plate (*Beckton Dickinson*, *Le Pont de Claix*, *France*). Sabouraud slants were incubated for 21 days at 30°C or 35°C and CHROMagar^TM^ *Candida* plate for 72h at 35°C. All sputa were divided into aliquots of 250 mg each and stored at -20°C for DNA extraction (Fig 1). This work was part of MucoBacMyco project, which was approved by an *ad hoc* Ethics Committee named Comité de Protection des Personnes d’Ile de France IX (N° CPP-IDF IX-12-011).

### DNA extraction protocols

All samples (250 mg aliquots) stored at -20°C were subjected to two different genomic DNA extraction protocols (Manual Powersoil® extraction -MPE- and Automated QIAsymphony extraction -AQE, see below). Negative extraction controls (250 μL DNA-free water) were processed in the same way as samples.

#### Manual Powersoil® extraction (MPE), MoBio PowerLyzer PowerSoil® DNA Isolation kit (QIAGEN, Carlsbad, USA)

The aliquots were pipetted into PowerLyzer® 0.1 mm Glass Bead Tubes and submitted to mechanical lysis: two 60-sec cycles at 6400 rpm on MagNA Lyser Instrument (Roche, Mannheim, Germany) and 1 min rest on ice between cycles. We then proceeded as per manufacturer’s instructions with minor adjustments: centrifugation of the Glass Bead Tubes for 5 mins instead of 30 secs (step 7 of the protocol, after mechanical lysis) and 5 mins waiting at room temperature (instead of 30 secs) before spinning at 10000g for 1 min (step 22) [6].

#### Automated QIAsymphony extraction (AQE), DSP DNA midi kit (QIAGEN, Carlsbad, USA)

The aliquots were diluted in 450 μL of ATL buffer (QIAGEN, Carlsbad, USA) and mixed with 1.4 mm ceramic MagNA Lyser green beads before mechanical lysis (as described above). They were centrifuged 3 mins at 10000g, and 450μL of supernatant was transferred to a new tube for enzymatic lysis (25μL of Proteinase K (Thermo Fisher Scientific, *Courtaboeuf*, *France*), for 45 minutes, at 56°C). The aliquots were submitted to automated extraction on the QIAsymphony using DSP DNA midi kit as recommended by the manufacturer.

### Nucleic acid quantification

The evaluation of DNA quality, and especially of RNA significant contamination, was performed using agar electrophoresis of DNA samples (1% agarose gel, 100V 30-minute migration). Extracted DNAs were quantified using Thermo Scientific^TM^ Nanodrop^TM^ 2000 spectophotometer (Thermo Fisher Scientific, *Courtaboeuf*, *France*), and Quant-iT PicoGreen dsDNA Assay Kit (Invitogren, Carlsbad, USA).

### *Aspergillus* DNA detection by real-time PCR in spiked sputa

The DNA extracts of the spiked sputa were analysed for *Aspergillus* DNA detection using real-time PCR targeting the 28S region of *A*. *fumigatus* [24] and the mitochondrial DNA of *Aspergillus* sp [25]. PCR was performed on a StepOnePlus System (Applied Biosystems, Foster City, USA).

### PCR amplification and sequencing

Amplicon libraries targeting V1-V2 or V3-V4 16S hyper-variable regions for bacteria and ITS1 or ITS2 regions for fungi were prepared. The employed 16S primers (for V1-V2: primers 27 and 338; for V3-V4: primers 341 and 785 without degenerate nucleotides [7,26]) were those recommended in the 16S Metagenomic Sequencing Library Preparation guide [27] (http://emea.support.illumina.com). The ITS1 primers were ITS1F [28,29] and ITS2; the ITS2, primers were ITS3 and ITS4 [30], all after inclusion of the Illumina sequencing adapters [27]. PCR amplicons were generated using Veriti^™^ Thermal Cycler (Applied Biosystems, Foster City, USA) in the following conditions: 3 mins at 95°C denaturation; 35 cycles (94°C for 30 secs, 53°C for 30 secs, and 72°C for 30 secs), and 5 mins at 72°C final elongation before cooling at 4°C. Positive controls consisting in DNA from *Escherichia* and *Candida* and negative controls were used for V1-V2 and V3-V4 16S, ITS1 and ITS2 amplifications, respectively. Each sample was purified with Agencourt AMPure XP (Beckman Coulter, *Villepinte*, *France*) as described in the 16S Metagenomic Sequencing Library Preparation guide [27]. Each sample was run on Agilent 2100 Bioanalyzer (Agilent, Santa Clara, USA) using Agilent High Sensitivity DNA Kit (Agilent, Santa Clara, USA) following manufacturer’s instructions. PCR products were barcoded using Nextera XT Index Kit (Illumina, *Evry*, *France*). The index PCRs were performed as recommended in the 16S Metagenomic Sequencing Library Preparation guide: 3 mins at 95°C denaturation; 8 cycles (94°C for 30 secs, 53°C for 30 secs, and 72°C for 30 secs), and 5 mins at 72°C final elongation before cooling at 4°C. Barcoded PCR products were purified using Agencourt AMPure XP, analysed on Agilent 2100 Bioanalyzer and quantified with Quant-iT PicoGreen dsDNA Assay Kit. Samples were normalized to 4nM and pooled (5μl of each normalized sample) into a library. A sequencing PhiX control (Illumina, *Evry*, *France*) was prepared following manufacturer’s instructions. The libraries were sequenced using MiSeq Reagent Kit V3 (2 x 300 bp with the 600-cycle kit, Illumina, *Evry*, *France*) on Illumina MiSeq platform (Illumina, *Evry*, *France*), and resulted in 115,286; 392,274; 68044 and 196230 sequence reads for V1-V2, V3-V4 16S, ITS1, and ITS2, respectively. Raw data are available on Genbank NCBI NIH (SRA accession number: PRJNA548447; https://www.ncbi.nlm.nih.gov/sra/PRJNA548447).

### Taxonomic assignment, diversity and statistical analyses

The generated data were analysed with PyroMIC© (protected software, IDDN FR.001.400018.000.S.P.2014.000.31230) which contains a cleaned fungal and bacterial database created from NCBI sequences. Ultimately, only sequences of >100bp for ITS reads and >150bp for 16S reads, with ≥98.7% and ≥94.5% homology, respectively, and 0.0 e-value were considered for identification at species and genus level [31]. Accordingly, we only retained 93,451; 276,475; 65,112 and 191,349 sequence reads for V1-V2, V3-V4 16S, ITS1, and ITS2, respectively (Table 1).

**Table 1:** Results of the high-throughput sequencing (reads and assignment) of bacterial (V1-V2 and V3-V4 16S targets) and fungal (ITS1 and ITS2) targets of 2 sputa from 2 patients (P1, P2) extracted using 2 protocols^a^ (Automated QIAsymphony Extraction [AQE] with DSP DNA midi kit and Manual PowerSoil® Extraction [MPE]).

		Overall	P1 Reads *(%)*		P2 Reads *(%)*	
		Reads *(%)*	MPE	AQE	MPE	AQE
	**Reads assigned as bacteria**	**93,451**	**20,908**	**10,366**	**35,055**	**27,122**
**16S,**	– At species level	44,032 *(47*.*1)*	20,379 *(97*.*5)*	10,021 *(96*.*7)*	7,852 *(22*.*4)*	5,780 *(21*.*3)*
**V1-V2**	– At genus level only	48,521 *(51*.*9)*	301 *(1*.*4)*	212 *(2*.*0)*	26,797 *(76*.*4)*	21,211 *(78*.*2)*
	– Unidentified bacteria	898 *(1)*	228 *(1*.*1)*	133 *(1*.*3)*	406 *(1*.*2)*	131 *(0*.*5)*
	Nb of taxa^b^	25	18	17	12	12
	**Reads assigned as bacteria**	**276,475**	**61,552**	**32,669**	**113,963**	**68,291**
**16S,**	– At species level	220,980 *(79*.*9)*	60,339 *(98*.*0)*	31,697 *(97*.*0)*	78,942 *(69*.*3)*	50,002 *(73*.*2)*
**V3-V4**	– At genus level only	49,011 *(17*.*7)*	908 *(1*.*5)*	713 *(2*.*2)*	31,164 *(27*.*3)*	16,226 *(23*.*8)*
	– Unidentified bacteria	6484 *(2*.*3)*	305 *(0*.*5)*	259 *(0*.*8)*	3857 *(3*.*4)*	2063 *(3*.*0)*
	Nb of taxa^b^	41	32	29	20	15
	**Reads assigned as fungi**	**65,112**	**20,926**	**22,832**	**6,940**	**14,414**
	– At species level	63,477 *(97*.*5)*	20,203 *(96*.*5)*	22,667 *(99*.*3)*	6,294 *(90*.*7)*	14,313 *(99*.*3)*
**ITS1**	– At genus level only	1,616 *(2*.*5)*	711 *(3*.*4)*	160 *(0*.*7)*	646 *(9*.*3)*	99 *(0*.*7)*
	– Unidentified fungi	19 *(0*.*0)*	12 *(0*.*1)*	5 *(0*.*0)*	0 *(0*.*0)*	2 *(0*.*0)*
	Nb of taxa^b^	5	5	5	4	3
	**Reads assigned as fungi**	**191,349**	**34,033**	**56,206**	**23,279**	**77,831**
	– At species level	154,347 *(80*.*7)*	419 *(1*.*2)*	55,566 *(98*.*9)*	21,535 *(92*.*5)*	76,827 *(98*.*7)*
**ITS2**	– At genus level only	36,982 *(19*.*3)*	33,609 *(98*.*8)*	635 *(1*.*1)*	1,738 *(7*.*5)*	1,000 *(1*.*3)*
	– Unidentified fungi	20 *(0*.*0)*	5 *(0*.*0)*	5 *(0*.*0)*	6 *(0*.*0)*	4 *(0*.*0)*
	Nb of taxa^b^	10	8	7	7	7

a- Methods used the same starting quantities of sample and final volumes were equal.

b- At genus level.

Rarefaction curves were computed to evaluate the quality of the taxonomic diversity assessment. The fungal and bacterial diversity patterns of samples were compared, at genus level, with the diversity patterns of negative controls for each amplification target. In case of similar profiles, contamination of samples by environmental sources was suspected and the samples were analysed one more time. Diversity indexes (Shannon, Shannon evenness, Simpson, Inverse Simpson, and Chao1) were calculated to compare the homogeneity of the samples in terms of bacterial and fungal microbiota composition. The community structure and the relative abundances of the bacterial and fungal taxa were compared according to the extraction protocol and the amplification targets using SHAMAN software (http://shaman.pasteur.fr/) [32]. The analyses of bacterial and fungal diversities were performed at genus level, as 16S/ITS amplicon sequencing is not sensitive enough to obtain precise identification of all micro-organisms at species level. Normalization of read counts was performed using DESeq2 normalization method [33]. The generalized linear model (GLM) implemented in DESeq2 R package was used to detect differences in abundance of taxa between the extraction protocols (MPE vs. AQE), the 16S (V1-V2 vs. V3-V4), and the ITS (ITS1 vs. ITS2) targets. GLM was computed to include the patient, the extraction protocol, and the amplification target as main effects, in addition to interaction between the extraction and the amplification target. This interaction was useful to model the pairing between successive measurements coming from the same individual. Resulting *P*-values were adjusted according to Benjamini and Hochberg procedure. Wilcoxon rank-sum test (α = 0.05) was used to compare DNA yield by the extraction protocol type.

## Results

### Influence of extraction protocol on DNA quantity and quality

Beginning with the same amount (250 ± 10 mg) of respiratory samples, we obtained DNA yields ranging from 0 to 37 ng/ml with MPE and from 15 to 411 ng/ml with AQE. For two samples (P4 and P5), no DNA was extracted with MPE method. On average, AQE method yielded double strand DNA of 4.5 times (fold-range [1.3–11]) higher than MPE (p-value = 0.03), within a shorter technical time (~30 min for AQE vs. ~90 min for MPE), and an equivalent cost. Regarding the quality and purity of the DNA, we did not observe significant RNA contamination using agar electrophoresis of DNA samples. AQE method produced higher rates of single-strand DNA than MPE. However, the 260/280 nm absorbance ratio was similar for both extraction protocols (p-value = 0.40, S1 Table).

### Bacterial and fungal culture of the two spiked sputa

The bacterial culture of the two spiked sputa was performed during routine analysis. For both patients, the culture revealed commensal flora without significant pathogens (i.e. pathogens ≥10^7^ CFU/ml as defined in REMIC [34]).

**Fig 2:**
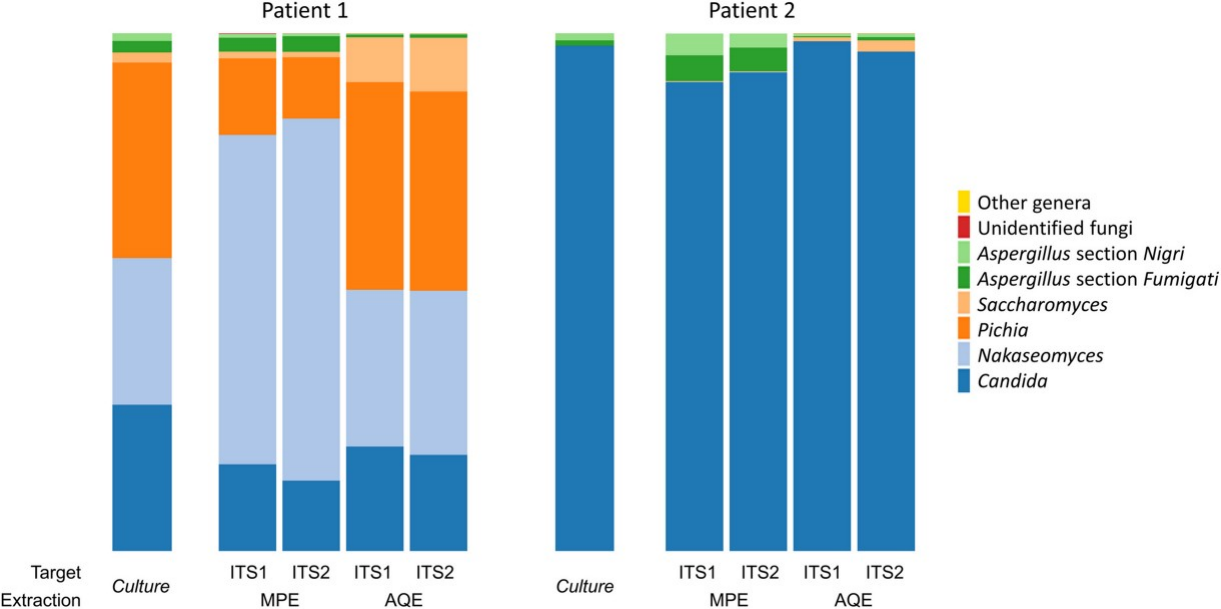
Taxa plots summarizing the relative abundances of fungal genera or sections identified in the respiratory samples of 2 patients (P1 and P2) using culture and ITS targeted amplicon sequencing. The sputa of P1 and P2 were spiked with 10^5^ CFU/ml of *Aspergillus* section *Nigri* and *Aspergillus* section *Fumigati*. For ITS targeted amplicon sequencing analysis, DNA was extracted from the respiratory samples using 2 protocols: the automated QIAsymphony extraction [AQE] with DSP DNA midi kit, and the manual PowerSoil® MoBio extraction [MPE]; and amplified by ultra-deep sequencing (MiSeq®, Illumina) using two ITS targets (ITS1 and ITS2). Methods used the same starting quantities of samples and the final volumes were equal. Metagenomic analysis revealed 5 extremely low-represented genera (overall relative abundance < 0.1% for each taxon) which were gathered in “other genera” category: *Rhizophlyctis*, *Schizophyllum*, *Hanseniaspora*, *Penicillium*, and *Inocutis*.

The fungal culture of the two spiked sputa (P1 and P2) revealed the presence of six fungi for P1 (Fig 2): 10^4^CFU/ml of *Candida krusei* (teleomorph name: *Pichia kudriavzevii)*; 7x10^3^ CFU/ml of *Candida albicans*; 7x10^3^ of *Candida glabrata* (of *Nakaseomyces* genus); 5x10^2^ CFU/ml of *Saccharomyces cerevisiae*; 6x10^2^ CFU/ml of *Aspergillus* section *Fumigati*, and 4x10^2^ CFU/ml of *Aspergillus* section *Nigri*. Only three fungi were identified for P2: 2x10^4^ CFU/ml of *C*. *albicans*; 2x10^2^ CFU/ml of *Aspergillus* section *Fumigati*, and 3x10^2^ CFU/ml *Aspergillus* section *Nigri* (Fig 2).

### *Aspergillus* DNA detection using qPCR

For the two spiked sputa (P1 and P2), the amount of *Aspergillus* DNA detected by 28S- and mt-qPCR was comparable (S2 Table) regardless of the extraction protocol.

### Bacterial diversity and community structure detected using V1-V2 and V4-V3 16S targeted amplicon sequencing

**Fig 3:**
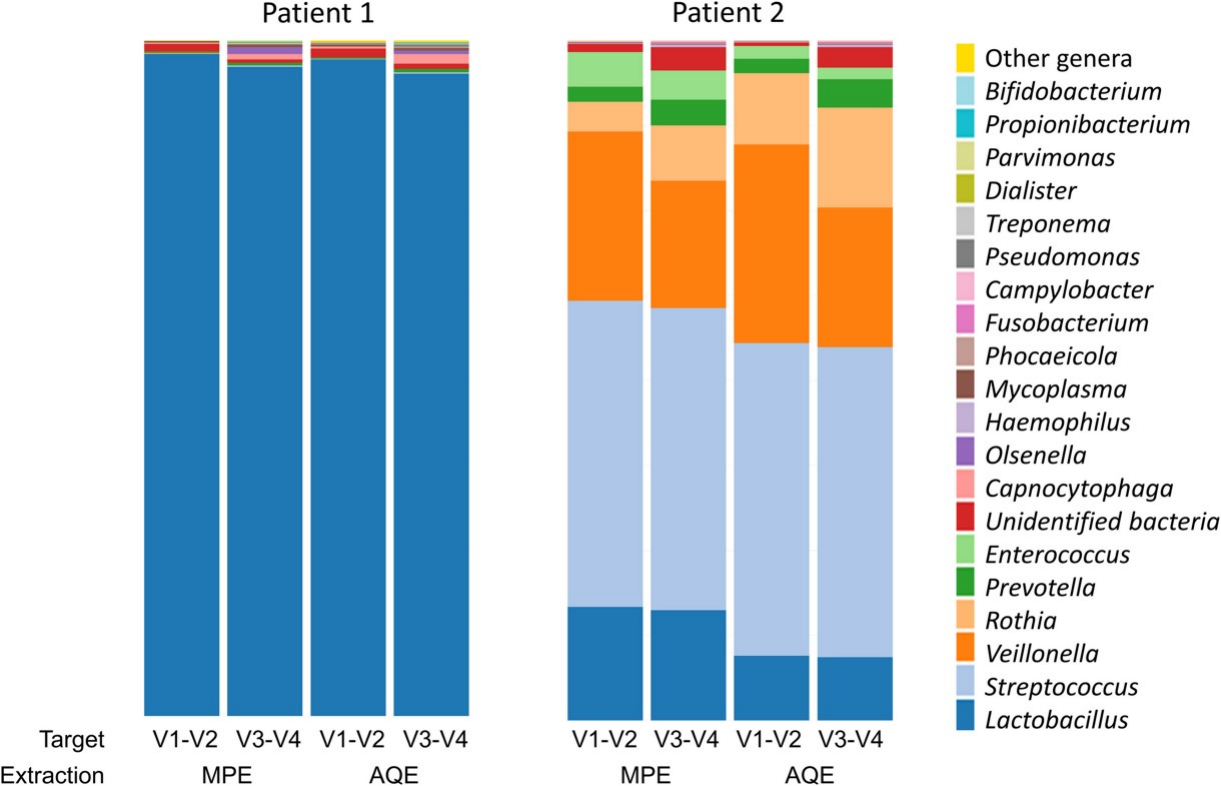
Taxa plots summarizing the relative abundances of bacterial genera identified in the respiratory samples of 2 patients (P1 and P2) using 16S targeted amplicon sequencing. DNA was extracted using 2 protocols: the automated QIAsymphony extraction [AQE] with DSP DNA midi kit, and the manual PowerSoil® MoBio extraction [MPE]; and amplified by ultra-deep sequencing (MiSeq®, Illumina) using two 16S targets (V1-V2 and V3-V4).

For P1 spiked-sputum, we analysed 31,274 paired-end reads using V1-V2 region (20,908 and 10,366 reads using MPE and AQE, respectively) and 94,221 reads using V3-V4 (61,552 and 32,669 with MPE and AQE, respectively) (Table 1). For P2 spiked-sputum, we analysed 62,177 reads using V1-V2 (35,055 and 27,122 reads using MPE and AQE, respectively), and 182,254 reads with V3-V4 (113,963 and 68,291 with MPE and AQE, respectively) (Table 1). For every event (2 patients x 2 extraction protocols x 2 amplification targets), the curves reached a plateau, indicating that the bacterial diversity had been satisfactorily detected (S1 Fig). The diversity patterns observed for every condition were different from negative controls and no sample had suffered from environmental contamination (S3 Table). Overall, 43 taxa were identified at genus level and one additional taxon (“Unidentified bacteria”) gathered unclassified reads. Even though a higher number of taxa were detected in P1’s samples (37 taxa *vs*. 21 in P2’s samples), P1 airway microbiota was less even than P2. Of more, P1 had one vastly predominant taxon (*Lactobacillus*, overall and per sample relative abundance is >95%), six minor taxa (*Streptococcus*, *Prevotella*, *Phocaeicola*, *Olsenella*, *Mycoplasma*, *Capnocytophaga;* overall relative abundances range from 0.1% to 1%), and 30 extremely low-represented taxa (overall relative abundance < 0.1%) (Fig 3). In contrast, P2 had a more even airway microbiota of six major taxa (*Lactobacillus*, *Streptococcus*, *Veillonella*, *Rothia*, *Prevotella*, *Enterococcus*; overall and per sample relative abundance is >1%), three minor taxa (*Haemophilus*, *Fusobacterium*, *Campylobacter*; overall relative abundances vary from 0.1% to 1%), and 12 extremely low-represented taxa (overall relative abundance <0.1%) (Fig 3). Altogether, 16 bacterial taxa were found in both P1 and P2. Of those, *Lactobacillus* was identified as the only “core” taxon (> 1% abundance in every tested sample), and five major taxa were dominant in P2 (*Streptococcus*, *Veillonella*, *Rothia*, *Prevotella*, *Enterococcus*). If both methods were able to detect 6/6 major and 7/9 minor taxa, we noticed that with V3-V4 a bigger number of extremely low-represented taxa were detected (15 for P1 and 9 for P2). In contrast, only 2 extremely low-represented taxa were detected with V1-V2 exclusively. We also observed that 11 genera were detected with significantly higher relative abundances using V3-V4 than V1-V2 (*Prevotella*, *Phocaeicola*, *Lactobacillus*, and *Mycoplasma*, *p-values* <0.001; *Streptococcus and Campylobacter*, 0.001*<p-values*<0.01*; Fusobacterium*, *Pseudomonas*, *Olsenella*, *Treponema*, *Rothia*, 0.01<*p-values*<0.05; S2 Fig; S4 Table). Pooled Chao1 index was higher for V3-V4 (50.7) than for V1-V2 (29.7).

Methods used the same starting quantities of samples and the final volumes were equal. The category “Other genera” gathered 23 bacterial taxa associated with extremely low relative abundances (overall relative abundance < 0.1% for each taxon): *Parascardovia*, *Anaeroglobus*, *Actinomyces*, *Howardella*, *Staphylococcus*, *Alloprevotella*, *Pseudoramibacter*, *Melissococcus*, *Corynebacterium*, *Fretibacterium*, *Stenotrophomonas*, *Atopobium*, *Sphingomonas*, *Acinetobacter*, *Shuttleworthia*, *Acidaminococcus*, *Moraxella*, *Oribacterium*, *Delftia*, *Eubacterium*, *Lactococcus*, *Mogibacterium*, *Chelativorans*, and *Vagococcus*.

### Fungal diversity and community structure detected using ITS1 and ITS2 targeted amplicon sequencing

For P1, we analysed 43,758 paired-end reads using ITS1 region (20,926 and 22,832 reads using MPE and AQE, respectively), and 90,239 reads using ITS2 (34,033 and 56,206 with MPE and AQE, respectively) (Table 1). For P2, we analysed 21,354 reads using ITS1 (6,940 and 14,414 reads using MPE and AQE, respectively), and 101,110 reads with ITS2 (23,279 and 77,831 MPE and AQE, respectively) (Table 1). For every event (2 patients x 2 extraction protocols x 2 targets), the rarefaction curves reached a plateau, indicating that the fungal diversity had been satisfactorily detected (S1 Fig). The diversity patterns observed for every condition differed completely from negative controls and no sample had suffered from environmental contamination (S3 Table). Overall, ten taxa were identified at genus level and one taxon gathered unclassified fungal reads (“Unidentified fungi”). As for *Aspergillus* taxa, we were able to distinguish between *Aspergillus Fumigati* and *Aspergillus Nigri* sections. We detected 9/10 and 8/10 of the fungal genera in P1 and P2, respectively, and the 2 *Aspergillus* sections were detected in both patients (Fig 2). Setting aside the spiked *Aspergillus* taxa, we observed that P1 harbored a more diverse respiratory mycobiota with four major fungal genera (*Candida*, *Pichia*, *Nakaseomyces* and *Saccharomyces*; relative abundance of >1% for each event), and four extremely low-represented genera (*Rhizophlyctis*, *Schizophyllum*, *Hanseniaspora*, *Inocutis*; relative abundance of <0.1% for each event). P2 had a less even mycobiota with only one vastly predominant fungal taxon (*Candida*, >90% relative abundance for every event), 1 minor taxon (*Saccharomyces*, overall relative abundance between 0.1 and 1%), and 4 extremely low-represented genera (*Nakaseomyces*, *Pichia*, *Penicillium* and *Inocutis*; relative abundance <0.1%). We observed that 7/11 genera or sections were detected with significantly higher relative abundances using ITS2 compared with ITS1 (*Aspergillus* section *Fumigati* and *Saccharomyces*, *p-values*<0.01*; Aspergillus* section *Nigri*, *Candida*, *Pichia*, *Nakaseomyces*, *Saccharomyces*, *Inocutis*, 0.01<*p-values*<0.05; S3 Fig, S5 Table). Of note, 41% of the fungal reads were assigned at species level. In particular, 79.8% and 100% of *Candida* reads from P1 and P2, respectively, were identified with good sequence homology (>97%) as *Candida albicans*; 89.2% of *Pichia* reads from P1 were *Candida krusei*; 61.5% of *Nakaseomyces* reads from P1 were *Candida glabrata*, and 99.9% and 100% of *Saccharomyces* reads from P1 and P2, respectively, were *Saccharomyces cerevisiae*. Pooled Chao1 index was twice higher for ITS2 (15.4) than for ITS1 (7).

### Comparison of bacterial and fungal diversities as detected by extraction protocols, and comparison of fungal diversity as detected by ITS targeted amplicon sequencing and culture

The bacterial community diversity analysed at genus level and estimated by Shannon and Simpson alpha-diversity indexes was comparable for P1 and P2 for the four analysed events (S6 Table). The pooled Chao1 indexes comparing results of MPE vs. AQE methods were similar for bacteria (61.9 vs. 61.3, respectively). The fungal diversity at genus or section level estimated by Shannon and Simpson indexes was also comparable for the four analysed events (S6 Table). The pooled Chao1 indexes comparing overall results of MPE vs. AQE methods were similar for fungi (14.4 vs. 11.5, respectively).

**Fig 4:**
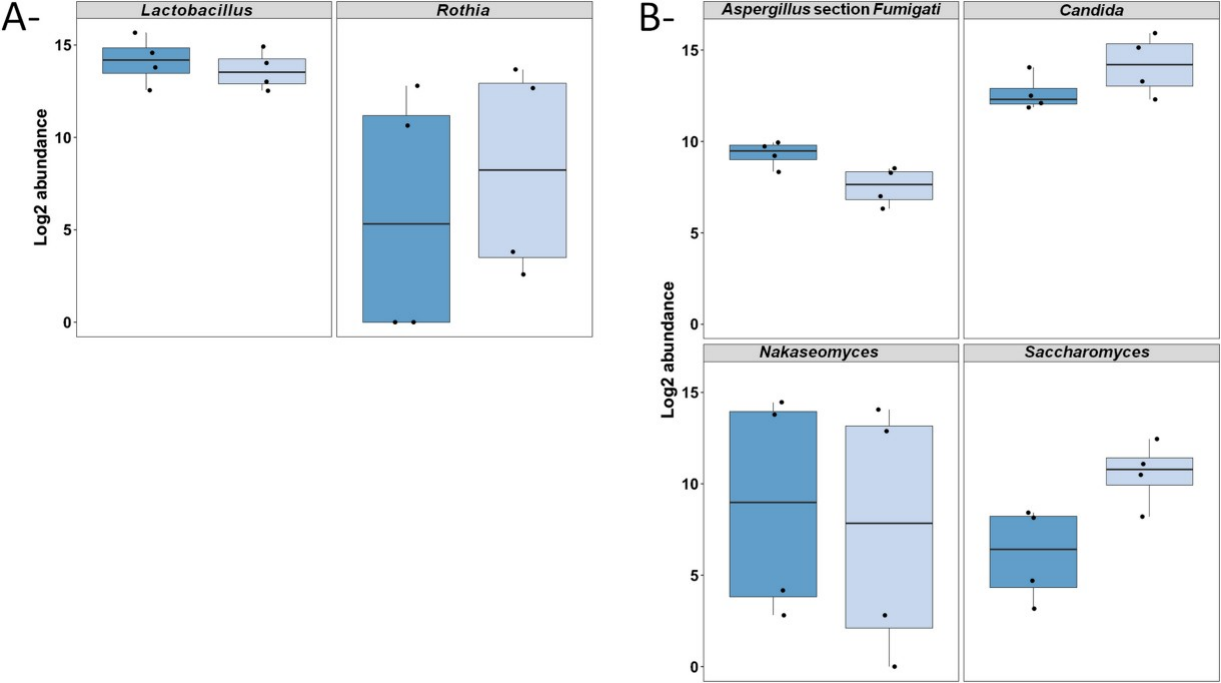
Boxplot of log2 abundances of bacterial (A) and fungal (B) taxa at genus or section level detected with significantly different abundances (*P*-value < 0.05) according to the employed extraction protocol. DNA was extracted either by manual PowerSoil® MoBio extraction -MPE- [dark blue] or automated QIAsymphony extraction -AQE- using DSP DNA midi kit [light blue].

Concerning the taxonomic diversity, both extraction protocols, MPE and AQE, failed in detecting 3 and 9/43 minor bacterial taxa and 1 and 2/11 minor fungal taxa, respectively. Regarding the relative abundances of taxa, the quantity detected was significantly impacted by extraction for 2 major bacterial genera (*Lactobacillus*, *p-value* = 0.008 and *Rothia*, *p-value* = 0.049) and 4 major fungal genera or sections (*Aspergillus* section *Fumigati* and *Saccharomyces*, *p-values*<0.001; *Candida*, *p-value* = 0.001; *Nakaseomyces*, *p-value* = 0.045) (Fig 4, S7 Table). The abundances of half of these taxa (*Lactobacillus*, *Aspergillus* section *Fumigati*, and *Nakaseomyces)* were higher with AQE, whereas the other half (*Rothia*, *Candida*, and *Pichia*) had their abundances decreased with AQE (Fig 4, S7 Table).

As for fungal diversity detected by ITS targeted amplicon sequecing and culture (Fig 2), we observed an overall agreement regarding major taxa since both methods detected 6/6 and 3/4 major taxa for P1 and P2, respectively. Only one major taxon, *Saccharomyces*, was detected in P2 samples by ITS amplicon sequencing but not by culture. All minor taxa (*Rhizophlyctis*, *Schizophyllum*, *Hanseniaspora*, *Penicillium*, *Inocutis*) were detected by sequencing only. The relative abundances of the major taxa detected by culture or ITS amplicon sequencing after AQE or MPE varied significantly. For P1, culture and AQE detected similar proportions of three major yeast taxa (*Candida*, *Nakaseomyces*, and *Pichia*). However, *Saccharomyces* and *Aspergillus* relative abundances were close in culture and MPE. For P2, the relative abundances of *Candida* and *Aspergillus* detected by AQE were closer to that observed in culture than MPE. But AQE detected a higher proportion of *Saccharomyces* compared with MPE or culture.

## Discussion

In the present study, we compared, simultaneously and under the same conditions, the impact of two DNA extraction protocols (manual PowerSoil MoBio® [12] and automated QIAsymphony extraction) on the diversity of respiratory bacterial and fungal microbiota assessed by 16S/ITS targeted amplicon sequencing using 2 bacterial (V1-V2 and V3-V4 16S) and 2 fungal (ITS1 and ITS2) amplification targets. Data of bacterial and fungal cultures were available and used to compare with the results of 16S/ITS targeted amplicon sequencing and *Aspergillus* qPCR. In our work, the use of automated extraction enhanced the DNA yield and quality with better reliability and shorter technical time than the manual protocol. The bacterial and fungal taxonomic diversity detected in respiratory samples was not highly influenced by the extraction protocol (low and moderate effect, respectively), whereas the amplification targets had a significant influence. A higher number of low density microbial communities were detected by V3-V4 16S and ITS2 targets.

Extraction had a significant influence on the yield of total DNA collected. AQE gave higher output (~4.5 times) of double-strand DNA with less heterogeneous results, in shorter technical time (~30 min) and an equivalent cost compared with MPE. We did not differentiate, as most authors, the human DNA yield from the microbiota DNA yield. However, it has been demonstrated that obtaining high total DNA yields is essential for bacterial targeted amplicon sequencing, and even more for fungal sequencing due to the low load of fungal genes in human microbiota [8,13,35]. Costea *et al* [8] compared 21 extraction protocols for bacterial intestinal metagenomic analysis and found that mechanical lysis and bead-beating were positively associated with bacterial diversity assessment, and that was of particular importance for efficient DNA extraction from Gram-positive bacteria. Equally, in our study, we were able to detect Gram-positive bacteria, e.g. *Streptococcus*, after AQE or MPE, indicating sufficient mechanical lysis. Regarding fungi, Huseyin *et al* [13] compared five extraction protocols for intestinal mycobiota analysis and observed that only the methods involving bead-beating steps enabled them to extract DNA in adequate quantities to provide further ITS-PCR products to undergo ultra-deep sequencing. Additionally, Vesty *et al* [14] compared four extraction protocols for fungal and bacterial oral 16S/ITS targeted amplicon sequecing and reported, as Sohrabi or Lazarevic did [36,37], that protocols comprising enzymatic lysis steps enhanced the extraction of DNA from the saliva. On the other hand, Rosenbaum *et al* [15] compared eight extraction protocols from oral rinse specimens for fungal and bacterial 16S/ITS targeted amplicon sequencing and found that DNA extraction methods had an important influence on overall DNA yield but no significant impact on oral microbiome composition. In their study, as in ours, the authors demonstrated that inter-individual variability drove wider fungal and bacterial diversity variations than did the extraction protocols [15].

We noticed that the choice of MPE or AQE protocol had only a moderate impact on the diversity and relative abundances of bacterial taxa detected in respiratory samples (2/43 taxa impacted in terms of relative abundance). Other authors [14,37,38] compared extraction protocols for the assessment of bacterial diversity in the upper respiratory tract microbiota and reported a high degree of congruence of the bacterial community structure irrespective of the employed extraction protocol. However, they noticed significant differences in the detection (presence/absence) of very-low abundance taxa and also in the relative abundances of a few major taxa. For instance, Lazarevic *et al* [37] and Biesbroek *et al* [38] showed that the relative abundances of *Firmicutes* and *Actinobacteria* were modified depending on the extraction method. Moreover, Lazarevic *et al* [37] observed that *Firmicutes* taxa were more highly represented in mechanically-treated samples, whereas *Actinobacteria* taxa were more highly represented in enzymatically-treated samples. In our study, the two bacterial taxa significantly impacted in terms of relative abundance were *Lactobacillus* (*Firmicutes*), which showed reduced abundance after AQE (despite the mechanical lysis), and *Rothia* (*Actinobacteria*) which had increased abundance after AQE (with an enzymatic lysis step). Overall, the moderate impact of the extraction protocols on the bacterial diversity in respiratory samples contrasts with results on samples from intestinal microbiota. In the gut, the extraction appears to highly influence the diversity and relative abundances of bacterial taxa [6,8]. This contrast might be explained by the lower diversity of the respiratory microbiota compared with the intestinal one, with maybe fewer bacterial taxa difficult to extract from respiratory samples. Interestingly, in our study, the use of AQE or MPE had a more significant impact on fungal communities (4/11 taxa impacted in term of relative abundance). Furthermore, we could not observe a recurrent pattern of decreased or increased relative abundances for these four taxa in relation with the protocol. To our knowledge, only Vesty *et al* [14] conducted a similar study comparing the fungal diversity of the upper respiratory tract and dental plaque in relation with extraction protocol. In their work, they did not document significant differences in the fungal communities from dental plaque but the plaque microbiota presented a very low diversity (>99% of *Candida*). In the saliva, 3/4 extraction protocols failed to yield an adequate number of reads to properly assess the fungal diversity. In our work, we also compared the fungal diversity assessed by ITS targeted amplicon sequencing using AQE or MPE versus culture results. In the literature, few studies have performed such comparison for fungal respiratory microbiota [2]. We confirm that amplicon sequencing detected more fungal taxa than culture. Taxa undetected by culture were mainly very-low represented taxa in addition to one major taxa, *Saccharomyces*. Concerning the relative abundances of major fungal taxa detected in our samples (*Aspergillus*, *Candida*, *Pichia*, *Nakaseomyces*, *and Saccharomyces*), the proportions detected by sequecing after AQE were closer to culture results, especially for *Candida*, *Pichia*, and *Nakaseomyce*s genera. However, we do not know whether culture results reflect the exact image of the composition/abundance of the respiratory fungal (or bacterial) microbiota.

The choice of the 16S or ITS primer sets used for targeted amplicon sequencing has a significant influence on the bacterial and fungal taxonomic diversity assessment. This is particularly visible for low density microbial communities. We detected more bacterial diversity, and particularly more extremely low-represented taxa using the 16S V3-V4 compared with V1-V2. Moreover, the relative abundances of 11/43 bacterial taxa were higher with the 16S V3-V4 target compared with V1-V2. Such results were somehow expected since V3-V4 primers had already been described by Klindworth *et al* as suitable for Illumina sequencing thanks to their high overall coverage associated with good domain specificity [26]. Clooney *et al* [7], in a study comparing three different high throughput sequencing technologies and two 16S region targets (V1-V2 and V4-V5), already observed that the primers they (like us) used for V1-V2 on Illumina MiSeq® were associated with the detection of a smaller number of species compared with other primers. For fungi, we detected more very low abundance fungal taxa with ITS2 than ITS1, with increased relative abundances in 7/11 fungal taxa using ITS2 compared with ITS1. The choice between ITS1 and ITS2 to study the fungal diversity in various microbiota remains undetermined to date. Different studies have compared both targets [22,39–42] but their results are not concordant. Wang *et al* [41] conducted a meta-analysis comparing both targets for eukaryotic analysis and discovered that ITS1 was superior to ITS2. They showed that ITS1 performed better in terms of successful amplification rates for a big number of eukaryotic species (due to lower GC content) and that the overall identification success rate was higher (especially for ferns, gymnosperms and ascomycetes). Inversely, other authors [22,39,40,43], who discussed this issue specifically for fungal analysis, did not demonstrate clear superiority of one target over the other. Blaalid *et al* [39] noticed comparable taxonomic composition at phylum and order level and some discrepancies on both sides (ITS1 and ITS2) at genus level. Bellemain *et al* [43] reported selective amplification biases associated with primers: the ITS1-F primer was biased towards basidiomycetes, while ITS2, ITS3, or ITS4 primers were biased towards ascomycetes [43]. Usyk *et al* [44] suggested that ITS1-F and ITS2 primers might not be appropriate for targeted amplicon sequencing and proposed new customized primers for ITS1 region. Ali *et al* [42], who worked specifically on respiratory specimen, found that amplification of ITS2 region was more accurate compared to ITS1. Finally, as both ITS targets have their advantages, several authors suggest using them concomitantly and then gather complementary information [22,40,43]. De Filippis *et al* [45] even hypothesised that targeting the ITS region only may lead to incorrect assessment of fungal communities. He suggested using other targets located on different genome regions in sequencing-based microbiota studies.

The microbiota profiles seen in our two patients were remarkably different, as often in sequencing-based microbiota analyses. P1 presented a very low bacterial diversity profile (one vastly predominant taxon, *Lactobacillus*) associated with a more diverse fungal profile (four major yeast taxa, *Candida*, *Pichia*, *Nakaseomyces*, and *Saccharomyces*). P2 had the inverse pattern: more bacterial diversity (six major taxa, *Streptococcus*, *Veillonella*, *Rothia*, *Prevotella*, *Lactobacillus*, and *Enterococcus*) and only one predominant fungal taxon (*Candida*). The major bacterial taxa identified in P2 (especially *Streptococcus*, *Veillonella*, and *Prevotella*) are known to be usual inhabitants of the upper respiratory tract of healthy humans [1,14,46,47], which is concordant with P2 status as a patient hospitalized for surgery without comorbidities. On the contrary, the very low-bacterial diversity profile of P1 was less usual, but this may be due to the immunocompromised status and the broad-spectrum antibacterial medicines received by that patient. Similar to the findings of other authors working on upper respiratory tract samples [14,19,48,49], we detected in both patients a predominance of yeasts: *Candida* (mainly *Candida albicans*), *Pichia* (mainly *Candida krusei*), *Nakaseomyces* (mainly *Candida glabrata*), and *Saccharomyces* (mainly *Saccharomyces cerevisiae*). However, and in contrast with data from the literature [14,48,49], we did not find reads assigned as *Malassezia*. This might be related to the extraction protocols we tested. Dupuy et al [49] explained that *Malassezia* species are known to have thick cell walls, hence the need for a harsh extraction protocol. The mechanical (with enzymatic or chemical lysis) used in AQE or MPE might not be strong enough to detect *Malassezia* reads. As for moulds taxa, we did not find significant genera other than *Aspergillus*, which were spiked in P1 and P2 sputa.

The main limitation of our study was the small number of samples analysed and the fact that we did not perform technical replications for the different conditions tested. However, in the emerging field of combined bacterial and fungal airway microbiota analysis, data regarding technical issues associated with extraction protocols or choice of primer sets are still scarce [14,15] and our results might still provide valuable information for other authors.

Another difficulty experienced in our study was that we were not able to extract, quantify or purify specifically human vs. bacterial or fungal DNA. This fact may introduce a potential bias in the quantification of our libraries prior sequencing. The presence of host DNA might thus decrease the depth of bacterial or fungal reads’ sequencing. However, to our knowledge, no satisfactory methods are yet available to specifically extract, purify or even quantify microbial DNA vs. host DNA with good reliability. To overcome the potential challenges associated with this issue, different strategies have been implemented in microbiota studies. First, when designing a targeted amplicon sequencing analysis, it is important to adapt the number of samples sequenced in each run in order to get a good sequencing depth for every sample and especially for microbial reads. Second, the bioinformatics pipelines applied to the raw data obtained after sequencing include a filtering process consisting in the removal of almost all reads assigned as human DNA. Over the past years, these bioinformatics pipelines have greatly improved and filtering process is now very efficient and stringent to remove host DNA. The filtered data are afterwards clustered and assigned to microbial taxa and finally the relative abundances of microbial taxa are compared between samples. These bioinformatics procedures allow us to limit the potential biases associated with the presence of host contaminant reads in various quantity from one sample to another. Also, an accurate representation of the microbial diversity within a sample can be detected, provided that the overall yield of DNA extracted at the beginning of the procedure was high enough [8,13].

Given that technical issues can influence the understanding of microbial communities, selecting protocols suitable for the characterization of the sputa microbiota is necessary to allow inter-study comparisons. Although our study included a small number of subjects, we observed that the use of an automated protocol (such as QIAsymphony) including both mechanical and enzymatic lysis enhanced the DNA extraction yield with more reliability and less technical time. Otherwise, the choice of extraction did not highly influence the bacterial or fungal taxonomic diversity detected in respiratory samples (low and moderate effect, respectively). In contrast, the choice of primer sets for targeted amplicon sequencing significantly influenced the bacterial and fungal diversity detected. In particular, we observed that V3-V4, 16S and ITS2 targets allowed to detect an increased number of low density microbial communities. Finally, for further 16S/ITS targeted amplicon sequencing on respiratory bacterial and fungal microbiota, we will choose to use the above described automated protocol and prefer the ITS2 target or, if possible, combine ITS1 and ITS2.

## Supporting information

S1 Fig Rarefaction curves of bacterial (A) and fungal (B) diversity identified from total DNA extracted from respiratory samples of 2 patients (P1, P2) using two extraction protocols (Automatic QIAsymphony Extraction [AQE, blue] with DSP DNA midi kit and Manual PowerSoil® Extraction [MPE, red]) and targeting 2 16S regions for bacterial analysis (V1-V2, solid line; V3-V4, dotted line) and 2 ITS regions for fungal analysis (ITS1, solid line; ITS2, dotted line).
(TIF)

S2 Fig Boxplot of log2 abundances of bacterial genera detected with significantly different abundances (*P*-value < 0.05) when amplified using either V1-V2 16S target [dark blue] or V3-V4 16S target [light blue].
(TIF)

S3 Fig Boxplot of log2 abundances of fungal genera or sections detected with significantly different abundances (*P*-value < 0.05) when amplified using either ITS1 target [dark blue] or ITS2 target [light blue].
(TIF)

S1 Table Results of DNA extractions from 6 respiratory samples (taken from 6 patients, P1 to P6) using two extraction protocols (Automated QIAsymphony Extraction [AQE] with DSP DNA midi kit and Manual PowerSoil® Extraction [MPE]). The DNA was quantified (ng/ml) using Picogreen® dosage and its quality was determined using 260/280 purity ratio (Nanodrop®).
(DOCX)

S2 Table Semi-quantitative detection of *Aspergillus* DNA extracted using two extraction protocols from respiratory samples of 2 patients (P1, P2). The extraction protocols used were the Automated QIAsymphony Extraction [AQE] with DSP DNA midi kit, and the Manual PowerSoil® Extraction [MPE]. The 2 sputa were spiked with 10^5^ conidia/ml of *Aspergillus* section *Fumigati* and *Aspergillus* section *Nigri*.
(DOCX)

S3 Table Diversity profiles (expressed in relative abundances at genus level) of negative controls according to sequencing amplification targets (16S V1-V2, 16S V3-V4, ITS1 and ITS2). (DOCX)

S4 Table Abundance Fold Change (expressed as log2 Fold Change) of bacterial genera significantly different (P-value < 0.05) with regard to the employed 16S target of amplification (V1-V2 vs. V3-V4). (DOCX)

S5 Table Abundance Fold Change (expressed as log2 Fold Change) of fungal genera or sections significantly different (P-value < 0.05) with regards to the used ITS target of amplification (ITS1 vs. ITS2). (DOCX)

S6 Table Alpha diversity measurements (Shannon, Simpson, and Chao1 indexes) for bacterial (A) and fungal (B) targeted amplicon sequencing analyses performed on respiratory samples of 2 patients (P1 and P2). DNA was extracted using two extraction protocols (Automated QIAsymphony Extraction [AQE] with DSP DNA midi kit, and Manual PowerSoil® Extraction [MPE]). Amplification targets used were VI-V2 and V3-V4 for bacterial analysis and ITS1 and ITS2 for fungal analysis.
(DOCX)

S7 Table Abundance Fold Change (expressed as log2 Fold Change) of bacterial (A) and fungal (B) taxa significantly different (P-value < 0.05) with regard to the used extraction protocol (manual PowerSoil® MoBio extraction [MPE] vs. automated QIAsymphony extraction [AQE]).
(DOCX)
