# Supplementary figures and images for: Combined bacterial and fungal targeted amplicon sequencing of respiratory samples: Does the DNA extraction method matter?
Source: PLoS One. 2020 Apr 28;15(4):e0232215. doi: 10.1371/journal.pone.0232215 (PMC7188255; doi:10.1371/journal.pone.0232215)

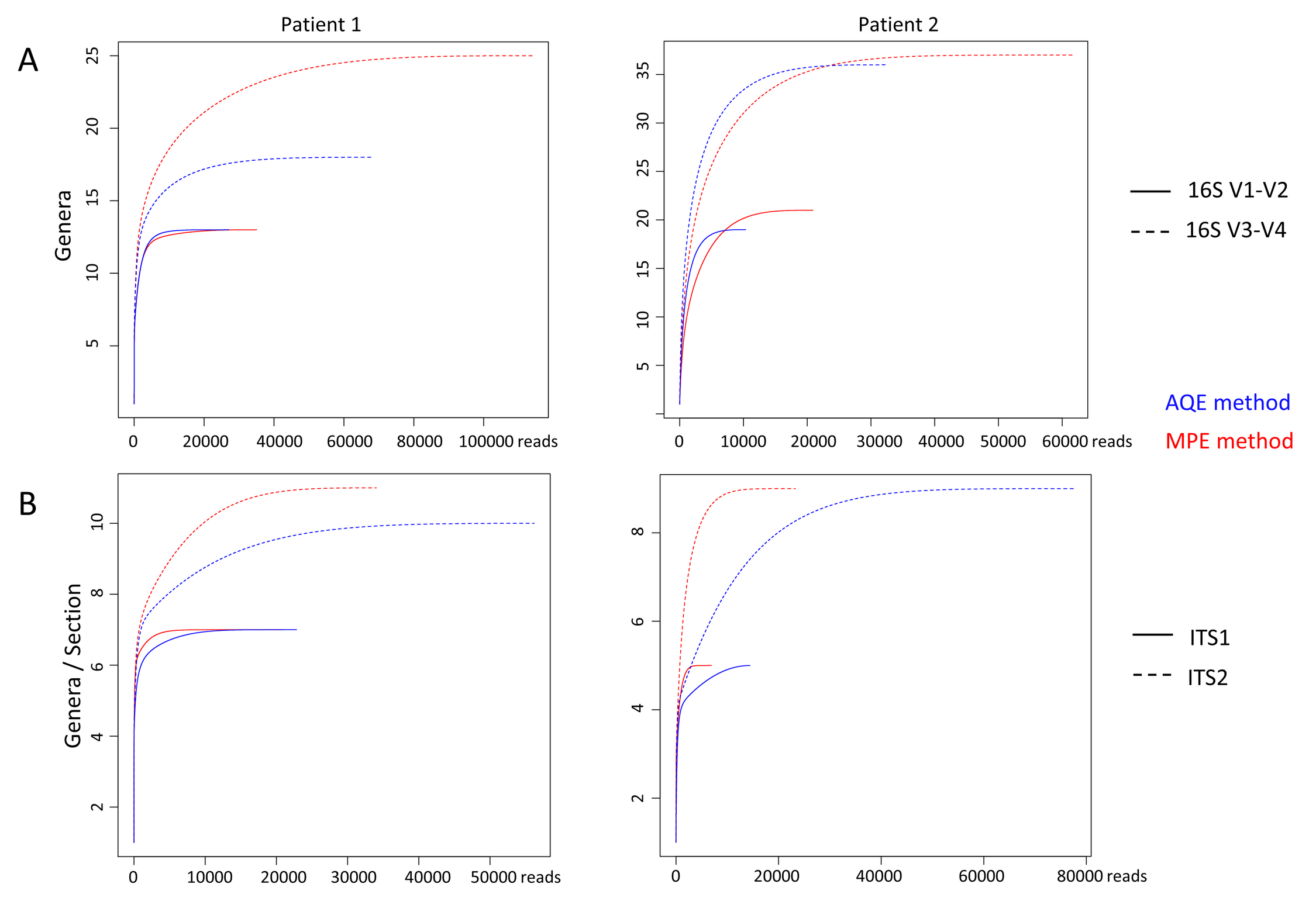

Supplement: S1 Fig — Rarefaction curves of bacterial (A) and fungal (B) diversity identified from total DNA extracted from respiratory samples of 2 patients (P1, P2) using two extraction protocols (Automatic QIAsymphony Extraction [AQE, blue] with DSP DNA midi kit and Manual PowerSoil® Extraction [MPE, red]) and targeting 2 16S regions for bacterial analysis (V1-V2, solid line; V3-V4, dotted line) and 2 ITS regions for fungal analysis (ITS1, solid line; ITS2, dotted line). (TIF) [file pone.0232215.s001.tif]

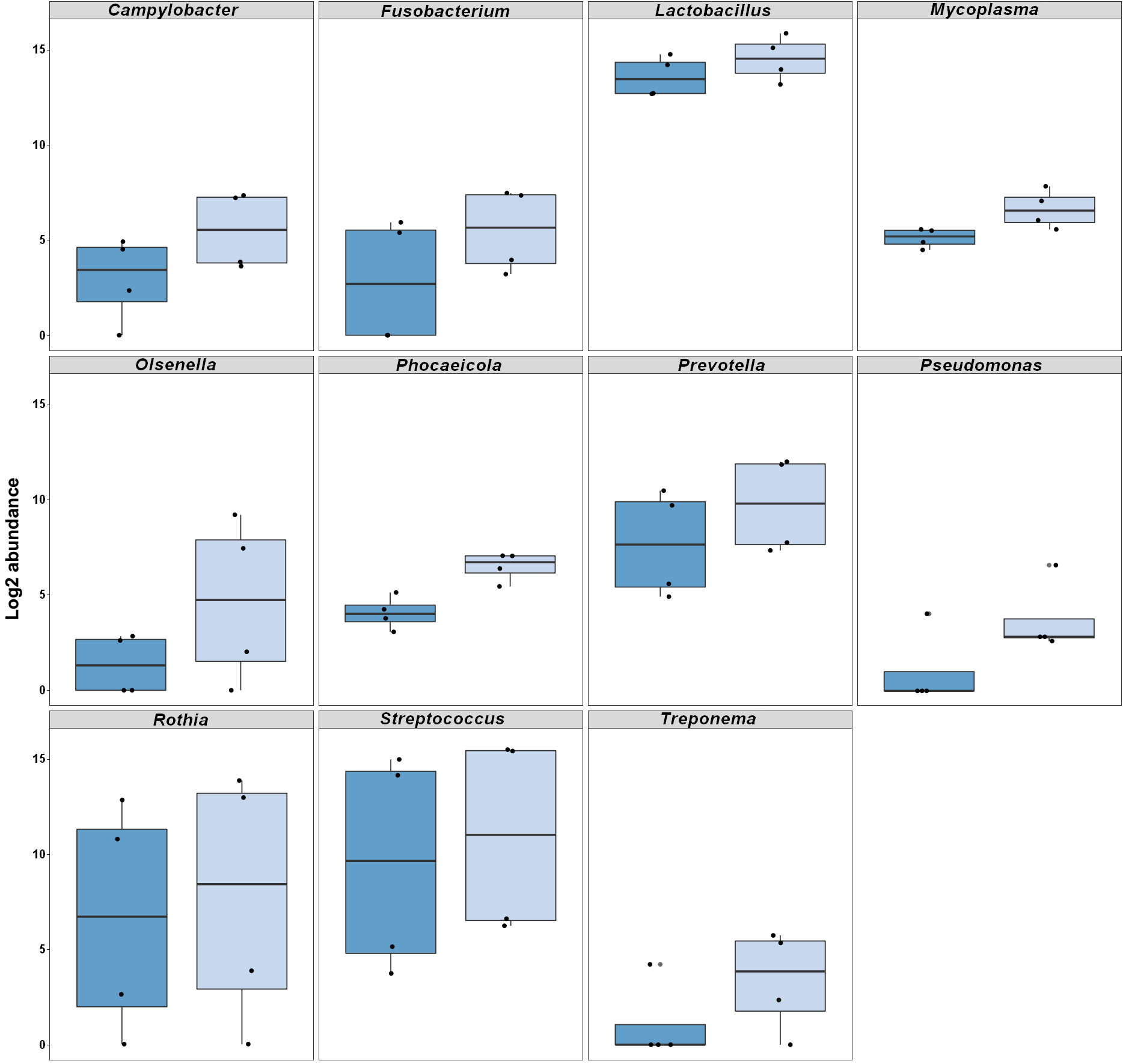

Supplement: S2 Fig — Boxplot of log2 abundances of bacterial genera detected with significantly different abundances (P-value < 0.05) when amplified using either V1-V2 16S target [dark blue] or V3-V4 16S target [light blue]. (TIF) [file pone.0232215.s002.tif]

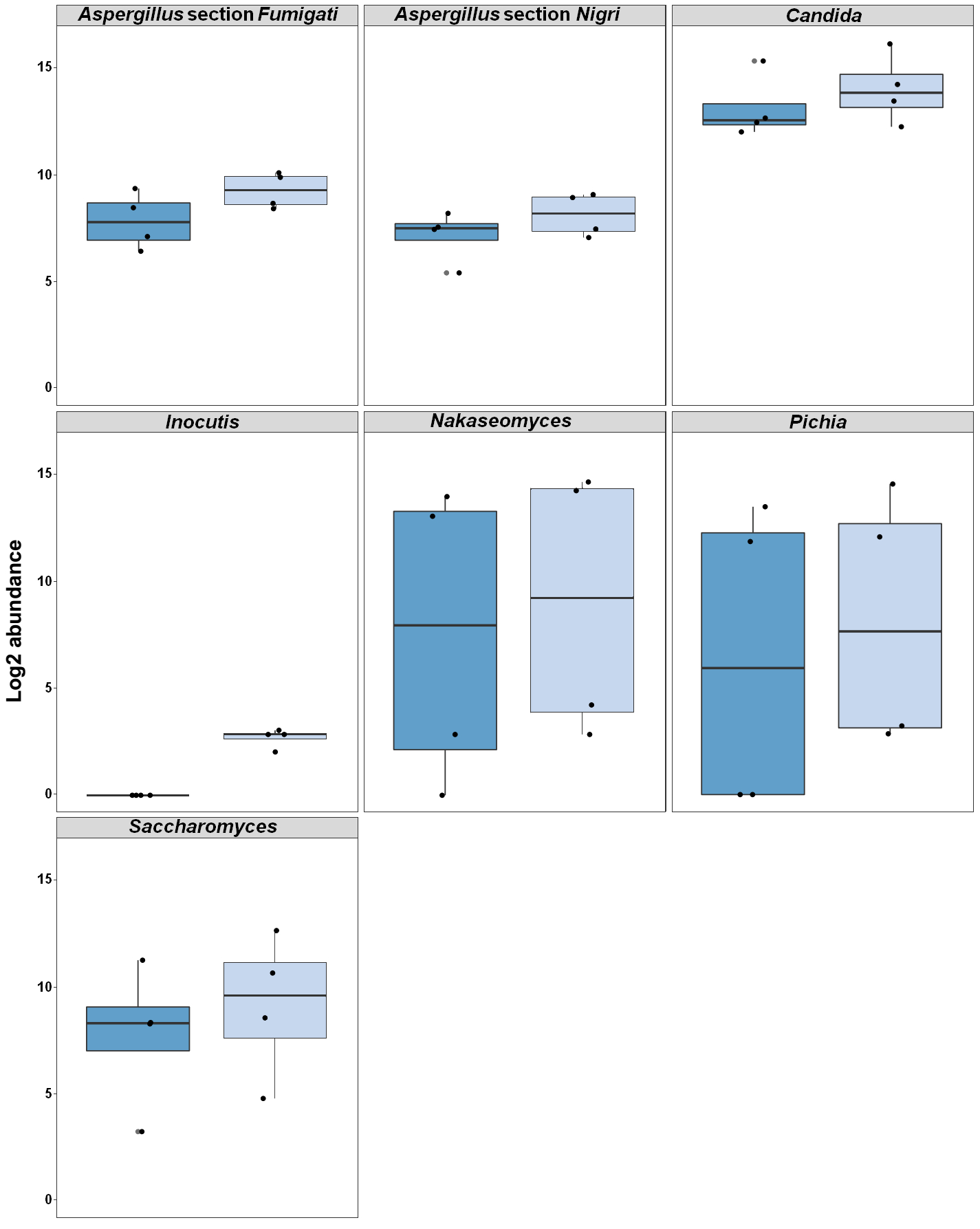

Supplement: S3 Fig — Boxplot of log2 abundances of fungal genera or sections detected with significantly different abundances (P-value < 0.05) when amplified using either ITS1 target [dark blue] or ITS2 target [light blue]. (TIF) [file pone.0232215.s003.tif]
