# Supplementary material for: Combined bacterial and fungal targeted amplicon sequencing of respiratory samples: Does the DNA extraction method matter?
Source: PLoS One. 2020 Apr 28;15(4):e0232215. doi: 10.1371/journal.pone.0232215 (PMC7188255; doi:10.1371/journal.pone.0232215)
Supplement: S1 Table — The DNA was quantified (ng/ml) using Picogreen® dosage and its quality was determined using 260/280 purity ratio (Nanodrop®). (DOCX) [file pone.0232215.s004.docx]

**S1 Table. Results of DNA extractions from 6 respiratory samples (taken from 6 patients, P1 to P6) using two extraction protocols (Automated QIAsymphony Extraction [AQE] with DSP DNA midi kit and Manual PowerSoil® Extraction [MPE]).** The DNA was quantified (ng/ml) using Picogreen® dosage and its quality was determined using 260/280 purity ratio (Nanodrop®).

|  | MPE method | | AQE method | | Ratio DNA AQE/MPE |
| --- | --- | --- | --- | --- | --- |
|  | DNA  ng/µl | 260/280  ratio | DNA  ng/µl | 260/280  ratio |  |
| P1 | 11.8 | 1.95 | 15.2 | 2.34 | 1.3 |
| P2 | 15.9 | 1.90 | 44.9 | 1.34 | 2.8 |
| P3 | 37.2 | 1.80 | 410.6 | 1.74 | 11.0 |
| P4 | 0.9 | 1.11 | 111.7 | 1.89 | 124.1 |
| P5 | 1.9 | 1.60 | 31.4 | 1.99 | 16.5 |
| P6 | 8.6 | 1.68 | 21.8 | 2.15 | 2.5 |
