# Supplementary material for: Combined bacterial and fungal targeted amplicon sequencing of respiratory samples: Does the DNA extraction method matter?
Source: PLoS One. 2020 Apr 28;15(4):e0232215. doi: 10.1371/journal.pone.0232215 (PMC7188255; doi:10.1371/journal.pone.0232215)
Supplement: S2 Table — The extraction protocols used were the Automated QIAsymphony Extraction [AQE] with DSP DNA midi kit, and the Manual PowerSoil® Extraction [MPE]. The 2 sputa were spiked with 105 conidia/ml of Aspergillus section Fumigati and Aspergillus section Nigri. (DOCX) [file pone.0232215.s005.docx]

**S2 Table. Semi-quantitative detection of *Aspergillus* DNA extracted using two extraction protocols from respiratory samples of 2 patients (P1, P2).** The extraction protocols used were the Automated QIAsymphony Extraction [AQE] with DSP DNA midi kit, and the Manual PowerSoil® Extraction [MPE]. The 2 sputa were spiked with 10^5^ conidia/ml of *Aspergillus* section *Fumigati* and *Aspergillus* section *Nigri*.

| Patient | Extraction method | 28S DNA (Ct) | Mitochondrial *Aspergillus* DNA (Ct) |
| --- | --- | --- | --- |
| P1 | MPE | 22.7 | 23.8 |
| P2 | MPE | 23.2 | 24.4 |
| P1 | AQE | 22.4 | 23.6 |
| P2 | AQE | 23.2 | 23.9 |
