# Supplementary material for: Combined bacterial and fungal targeted amplicon sequencing of respiratory samples: Does the DNA extraction method matter?
Source: PLoS One. 2020 Apr 28;15(4):e0232215. doi: 10.1371/journal.pone.0232215 (PMC7188255; doi:10.1371/journal.pone.0232215)
Supplement: S3 Table — (DOCX) [file pone.0232215.s006.docx]

**S3 Table**. **Diversity profiles (expressed in relative abundances at genus level) of negative controls according to sequencing amplification targets (16S V1-V2, 16S V3-V4, ITS1 and ITS2).**

| Taxa detected | 16S V1-V2  Negative control (Relative abundances %) | Taxa detected | 16S V3-V4  Negative control (Relative abundances %) |
| --- | --- | --- | --- |
| *Pseudomonas* | 15.6 | *Cutibacterium* | 25.1 |
| *Propionibacterium* | 15.4 | *Streptococcus* | 12.5 |
| *Paracoccus* | 11.4 | *Delftia* | 12.4 |
| *Stenotrophomonas* | 9.1 | *Corynebacterium* | 7.4 |
| *Beijerinckia* | 5.8 | *Rhizobium* | 7.4 |
| *Herbaspirillum* | 5.1 | *Sphingomonas* | 5.8 |
| *Sphingomonas* | 4.6 | *Herbaspirillum* | 5.3 |
| *Enterococcus* | 3.9 | *Cloacibacterium* | 4.4 |
| *Lactococcus* | 3.6 | *Staphylococcus* | 4.1 |
| *Pelomonas* | 2.9 | *Cyanobacteria* | 2.9 |
| *Pseudonocardia* | 2.9 | *Stenotrophomonas* | 2.3 |
| *Clostridium* | 2.9 | *Lawsonella* | 2.1 |
| *Prevotella* | 2.1 | *Moraxella* | 1.6 |
| *Micrococcus* | 1.8 | *Acinetobacter* | 1.2 |
| *Lactobacillus* | 1.6 | *Actinomyces* | 1.1 |
| *Delftia* | 1.5 | *Hymenobacter* | 1 |
| *Mesorhizobium* | 1.5 | *Xanthobacteriacaea* | 0.9 |
| *Microbacterium* | 1.3 | *Pseudomonas* | 0.4 |
| *Streptococcus* | 1.3 |  |  |
| *Bacillus* | 1.1 |  |  |
| *Gordonia* | 1.1 |  |  |
| *Cyanobium* | 1.0 |  |  |
| *Ochrobactrum* | 0.9 |  |  |
| *Acinetobacter* | 0.9 |  |  |
| *Corynebacterium* | 0.6 |  |  |
|  |  |  |  |
| Taxa detected | ITS1 Negative control (Relative abundances %) | Taxa detected | ITS2 Negative control (Relative abundances %) |
| *Preussia* | 85.5 | *Sarocladium* | 70.2 |
| *Sarocladium* | 5.9 | Undefined *Eurotiales* | 29.5 |
| *Stereum* | 4.5 |  |  |
| *Malassezia* | 4.4 |  |  |
