# Supplementary material for: Combined bacterial and fungal targeted amplicon sequencing of respiratory samples: Does the DNA extraction method matter?
Source: PLoS One. 2020 Apr 28;15(4):e0232215. doi: 10.1371/journal.pone.0232215 (PMC7188255; doi:10.1371/journal.pone.0232215)
Supplement: S4 Table — (DOCX) [file pone.0232215.s007.docx]

**S4 Table. Abundance Fold Change (expressed as log2 Fold Change) of bacterial genera significantly different (P-value < 0.05) with regard to the employed 16S target of amplification (V1-V2 vs. V3-V4).**

| **Bacterial genera significantly different** | **baseMean** | **log2 Fold Change** | ***P*-value** |
| --- | --- | --- | --- |
| *Prevotella* | 1,198 | -1.02 | <0.001 |
| *Phocaeicola* | 54 | -1.26 | <0.001 |
| *Lactobacillus* | 19,804 | -0.46 | <0.001 |
| *Mycoplasma* | 73 | -0.76 | <0.001 |
| *Streptococcus* | 15,560 | -0.71 | 0.001 |
| *Campylobacter* | 46 | -1.38 | 0.003 |
| *Fusobacterium* | 54 | -2.35 | 0.015 |
| *Pseudomonas* | 15 | -2.75 | 0.015 |
| *Olsenella* | 91 | -2.35 | 0.026 |
| *Treponema* | 14 | -2.16 | 0.044 |
| *Rothia* | 3,541 | -0.85 | 0.048 |
