# Supplementary material for: Combined bacterial and fungal targeted amplicon sequencing of respiratory samples: Does the DNA extraction method matter?
Source: PLoS One. 2020 Apr 28;15(4):e0232215. doi: 10.1371/journal.pone.0232215 (PMC7188255; doi:10.1371/journal.pone.0232215)
Supplement: S5 Table — (DOCX) [file pone.0232215.s008.docx]

**S5 Table. Abundance Fold Change (expressed as log2 Fold Change) of fungal genera or sections significantly different (P-value < 0.05) with regards to the used ITS target of amplification (ITS1 vs. ITS2).**

| **Fungal genera/sections significantly different** | **baseMean** | **log2 Fold Change** | ***P*-value** |
| --- | --- | --- | --- |
| *Aspergillus* section *Fumigati* | 454 | -0.71 | 0.006 |
| *Saccharomyces* | 1,271 | -0.69 | 0.006 |
| *Inocutis* | 3 | -3.37 | 0.029 |
| *Nakaseomyces* | 7,673 | -0.75 | 0.044 |
| *Aspergillus* section *Nigri* | 232 | -0.49 | 0.046 |
| *Pichia* | 4,727 | -1.88 | 0.046 |
| *Candida* | 18,023 | -0.45 | 0.047 |
