# Supplementary material for: Combined bacterial and fungal targeted amplicon sequencing of respiratory samples: Does the DNA extraction method matter?
Source: PLoS One. 2020 Apr 28;15(4):e0232215. doi: 10.1371/journal.pone.0232215 (PMC7188255; doi:10.1371/journal.pone.0232215)
Supplement: S6 Table — Alpha diversity measurements (Shannon, Simpson, and Chao1 indexes) for bacterial (A) and fungal (B) targeted amplicon sequencing analyses performed on respiratory samples of 2 patients (P1 and P2). DNA was extracted using two extraction protocols (Automated QIAsymphony Extraction [AQE] with DSP DNA midi kit, and Manual PowerSoil® Extraction [MPE]). Amplification targets used were VI-V2 and V3-V4 for bacterial analysis and ITS1 and ITS2 for fungal analysis. (DOCX) [file pone.0232215.s009.docx]

**S6 Table. Alpha diversity measurements (Shannon, Simpson, and Chao1 indexes) for bacterial (A) and fungal (B) metagenomic analyses performed on respiratory samples of 2 patients (P1 and P2).** DNA was extracted using two extraction protocols (Automated QIAsymphony Extraction [AQE] with DSP DNA midi kit, and Manual PowerSoil® Extraction [MPE]). Amplification targets used were VI-V2 and V3-V4 for bacterial analysis and ITS1 and ITS2 for fungal analysis.

|  | **P1** | | | | **P2** | | |
| --- | --- | --- | --- | --- | --- | --- | --- |
| **A - Bacterial diversity at genus level** | **Shannon** | **Simpson** | **Chao1** | **Shannon** | | **Simpson** | **Chao1** |
| MPE ; V1-V2 | 0.15 | 0.04 | 17 | 1.47 | | 0.71 | 13 |
| MPE ; V3-V4 | 0.26 | 0.08 | 25 | 1.61 | | 0.73 | 17 |
| AQE ; V1-V2 | 0.19 | 0.06 | 18 | 1.38 | | 0.68 | 13 |
| AQE ; V3-V4 | 0.33 | 0.10 | 29 | 1.53 | | 0.71 | 15 |
| **B - Fungal diversity at species/section level** | **Shannon** | **Simpson** | **Chao1** | **Shannon** | | **Simpson** | **Chao1** |
| MPE ; ITS1 | 1,06 | 0,54 | 7 | 0,39 | | 0,18 | 5 |
| MPE ; ITS2 | 0,96 | 0,48 | 9 | 0,34 | | 0,14 | 9 |
| AQE ; ITS1 | 1,30 | 0,70 | 7 | 0,09 | | 0,03 | 5 |
| AQE ; ITS2 | 1,33 | 0,71 | 6 | 0,19 | | 0,07 | 6 |
