# Supplementary material for: Combined bacterial and fungal targeted amplicon sequencing of respiratory samples: Does the DNA extraction method matter?
Source: PLoS One. 2020 Apr 28;15(4):e0232215. doi: 10.1371/journal.pone.0232215 (PMC7188255; doi:10.1371/journal.pone.0232215)
Supplement: S7 Table — Abundance Fold Change (expressed as log2 Fold Change) of bacterial (A) and fungal (B) taxa significantly different (P-value < 0.05) with regard to the used extraction protocol (manual PowerSoil® MoBio extraction [MPE] vs. automated QIAsymphony extraction [AQE]). (DOCX) [file pone.0232215.s010.docx]

**S7 Table. Abundance Fold Change (expressed as log2 Fold Change) of bacterial (A) and fungal (B) taxa significantly different (P-value < 0.05) with regard to the used extraction protocol (manual PowerSoil® MoBio extraction [MPE] vs. automated QIAsymphony extraction [AQE]).**

| 1. **Bacterial genera significantly different** | **baseMean** | **log2 Fold Change** | ***P*-value** |
| --- | --- | --- | --- |
| *Lactobacillus* | 19,804 | 0.26 | 0.008 |
| *Rothia* | 3,541 | -2.29 | 0.049 |
| 1. **Fungal genera or sections significantly different** | **baseMean** | **log2 FoldChange** | ***P*-value** |
| *Saccharomyces* | 1,271 | -2.24 | <0.001 |
| *Aspergillus* section *Fumigati* | 454 | 0.89 | <0.001 |
| *Candida* | 18,023 | -0.77 | 0.001 |
| *Nakaseomyces* | 7,673 | 0.72 | 0.045 |
